# Evaluating the Role of Probiotics in the Prevention and Management of Age-Related Diseases

**DOI:** 10.3390/ijms23073628

**Published:** 2022-03-26

**Authors:** Despoina E. Kiousi, Antonia Z. Kouroutzidou, Konstantinos Neanidis, Dimitrios Matthaios, Aglaia Pappa, Alex Galanis

**Affiliations:** 1 Department of Molecular Biology and Genetics, Faculty of Health Sciences, Democritus University of Thrace, 68100 Alexandroupolis, Greece; dkiousi@mbg.duth.gr (D.E.K.); toniakour@hotmail.com (A.Z.K.); 2 Oncology Department, 424 General Military Training Hospital, 56429 Thessaloniki, Greece; neanidis@hotmail.com; 3 Oncology Department, General Hospital of Rhodos, 85133 Rhodos, Greece; dimalexpoli@yahoo.com

**Keywords:** probiotics, aging, human health, cancer, bone diseases, neurodegenerative disorders

## Abstract

The human lifespan has been significantly increased due to scientific advancements in the management of disease; however, the health span of the aging population does not follow the same trend. Aging is the major risk factor for multimorbidity that is derived from the progressive loss of homeostasis, immunological and stem cell exhaustion, as well as exacerbated inflammation responses. Age-related diseases presenting with high frequencies include neurodegenerative, musculoskeletal, cardiovascular, metabolic diseases and cancer. These diseases can be co-morbid and are usually managed using a disease-specific approach that can eventually lead to polypharmacy, low medication adherence rates and undesired drug-drug interactions. Novel studies suggest targeting the shared biological basis of age-related diseases to retard the onset and manage their manifestations. Harvesting the anti-inflammatory and immunomodulatory capacity of probiotics to tackle the root cause of these diseases, could pose a viable alternative. In this article, a comprehensive review of the effects of probiotic supplementation on the molecular pathogenesis of age-related diseases, and the potential of probiotic treatments as preventative or alleviatory means is attempted. Furthermore, issues on the safety and efficiency of probiotic supplementation, as well as the pitfalls of current clinical studies are discussed, while new perspectives for systematic characterization of probiotic benefits on aged hosts are outlined.

## 1. Introduction

The percentage of people over the age of 65 years, is expected to double by 2050, as the pace of population ageing is continually accelerating [1]. Scientific advancements in the prevention and treatment of disease have prolonged human life span, leading to this phenomenon [2]. However, the increased life span is not accompanied by an elevated health span. Aging is considered as the strongest risk factor for multimorbidity and mortality [2]. Indeed, the continuum of age-related diseases includes cognitive, neurodegenerative, metabolic, cardiovascular, musculoskeletal conditions and cancer, that can ultimately lead to death. Additionally, elders commonly suffer from hearing and vision loss, frailty, decreased physical and psychological function that significantly affect quality of life [3]. For the management of these conditions a disease-specific approach is usually employed that can lead to polypharmacy, lower adherence rates, unwanted drug–drug interactions, and side effects [4]. Subsequently, the geroscience hypothesis proposes targeting the biological mechanisms that underly the onset and progression of disease, to tackle the multimorbidity that comes with age [5].

Aging is an irreversible biological process that can be defined using nine hallmarks: the deregulation of genetic, epigenetic, and immunological mechanisms (immunosenescence), as well as mitochondrial dysfunction, cell senescence, stem cell exhaustion and faulty nutrient sensing and intercellular signaling [6]. Cell senescence is a mechanism that ensures the homeostatic function of tissues and the prevention of carcinogenesis, that is triggered after multiple cell divisions and DNA damage [7]. The phenotype of senescent cells includes lack of proliferative potential, alteration of the euchromatin/heterochromatin ratio and epigenetic changes, as well as altered gene expression and protein accumulation. Importantly, these cells remain metabolically active, producing large quantities of cytokines and other immunostimulatory molecules [4]. The aberrant accumulation of these cells in tissues and organs during aging, stimulates local inflammation contributing to sub-clinical, low-grade inflammation [8]. Concomitantly, immune cell exhaustion that is derived by multiple antigen exposure, derails the clearance of these cells, also increasing susceptibility to infectious and autoimmune disease [9]. Apart from these nine hallmarks, novel studies suggest that gut microbiota dysbiosis could also play a significant role in the aging phenotype. The structure and function of gut microbiota remains relatively stable during adulthood; however, the aged microbiota presents a decline in microbial diversity, being more susceptible to the establishment of potential pathogenic species. In this context, several recent studies suggest that the aging microbiome presents abnormally high instability and heterogeneity between hosts, while specific microbial signatures of age-related diseases have started to be revealed [10]. Furthermore, increased gut permeability can amplify low-grade local and systemic inflammation, predisposing individuals to the onset of multimorbidity [11].

Two repurposed medications, rapamycin and metformin, primarily used for immunosuppressant purpose and for the treatment of type 2 diabetes, respectively, have shown experimental success in the delay of the aging phenotype and co-morbidity in preclinical and clinical studies, notwithstanding side effects [9]. Thus, alternative strategies are also being explored, such as the manipulation of the caloric intake and structure and function of the gut microbiome. In this context, caloric restriction has shown promising results on life- and health-span in animal studies, however, clinical studies often present contradictory results [9]. The high accessibility of the gut microbiome renders it a readily available target for manipulation. Several studies have proposed that the intake of probiotics can fine tune the gut microbial composition to more favorable structures in a host-specific manner [12,13]. The effect of ingested probiotics and the gut microbiome on the health span was first described by Elie Metchnikoff, who proposed that the consumption of fermented dairy products could prolong the life of Caucasian villagers [14]. Today, probiotics are defined as live microorganisms that can confer health benefits to the consumer, when administrated in adequate quantities [15]. These microorganisms can tolerate gastrointestinal passage and transiently colonize the hosts. Their ability to produce antimicrobial compounds or participate in trophic networks can induce structural changes in the microbiome [16]. Furthermore, their species-specific ability to interact with the host and induce immunomodulatory, anti-inflammatory and antioxidant effects or regulate cell cycle progression and cell death could suggest their ability to modulate the nine hallmarks of aging, and delay the onset, or alleviate the manifestations of age-related diseases.

**Figure 1:**
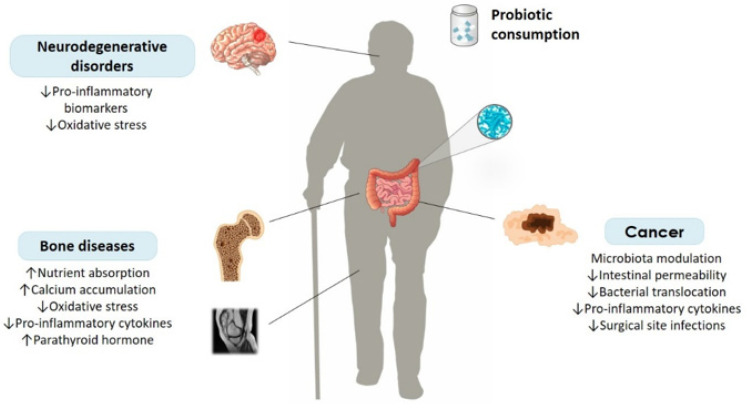
The effect of probiotic consumption on age-related intestinal and extraintestinal diseases. Probiotics can exert their beneficial actions by targeting the mechanisms of molecular pathogenesis of disease, such as inflammation, oxidative stress and hormonal signaling.

In this review article, we comprehensively present current clinical data on the effect of probiotic strains in the management of aging and its related conditions, in view of their molecular mechanisms of actions (Figure 1). Furthermore, we discuss the present and future of probiotic research in relation to aging and propose new strategies to refine supplementation regimens in order to maximize their efficacy and safety.

## 2. Age-Related Bone Diseases and Probiotics

The human skeleton possesses self-regeneration ability called remodeling, in which the old and damaged bone is removed and replaced with new intact tissue [17]. This regenerative process is performed thanks to the synergistic action of osteoclasts, osteoblasts, osteocytes, and bone lining cells within temporary anatomically distinct areas of skeleton, known as bone metabolic units (BMUs), that takes place throughout life [18]. However, the delicate balance between bone absorption and formation is usually disturbed during aging, favoring bone resorption over formation, resulting in several bone diseases, primarily osteoporosis [19]. Additionally, exhaustion of cell immunity and the induction of a pro-inflammatory microenvironment could lead to the development of autoimmune diseases of the bone. In this context, rheumatoid arthritis (RA) is a common chronic systemic inflammatory autoimmune disease that destroys bone and cartilage of joints and weakens tendons and ligaments [20].

### 2.1. Osteoporosis

Osteoporosis is a skeletal disorder characterized by low bone mass and microarchitectural deterioration of bone tissue, resulting in less bone tension or strength and increased risk of fragility fracture [21]. It is the most common age-related bone disease, and the main risk factor for fractures in the elderly population, that can lead to serious secondary health problems and even death [22]. Osteoporosis can be divided into two forms; primary osteoporosis, which includes postmenopausal osteoporosis (type I) and senile osteoporosis (type II), and secondary osteoporosis that is the outcome of underlying disease, or an effect of medication [23]. In this review, we focus on primary osteoporosis, as the most frequent age-related form of this disease. According to recent statistics from the International Osteoporosis Foundation, it is estimated that one in three women and one in five men over the age of 50 will develop osteoporotic fractures in their lifetime [24]. Primary osteoporosis is an outcome of bone homeostasis and hormonal imbalances and can also be promoted by pro-inflammatory events [21]. Recent studies have also shown that osteoporotic patients manifest gut microbial instability, characterized by elevated diversity that is correlated with decreased bone mass. Furthermore, qualitative differences can also be present, as it was observed that osteopenic and osteoporotic patients possess a higher abundance of the *Lachnoclostridium* and *Klebsiella* genera compared to healthy individuals [25]. Numerous treatments have been developed for the management of osteoporosis that aim at reversing bone loss and are mainly applied to patients with high fracture risk. Specifically, therapeutic interventions include drugs that inhibit bone resorption (anti-resorptive drugs), such as bisphosphonates [26,27] and parathyroid hormone (PTH), a stimulant of bone formation [28]. However, their administration is often accompanied by side effects, such as irritation of the upper gastrointestinal tract, atypical subtrochanteric femoral fractures, osteonecrosis of the jaw and even osteosarcoma [29]. Concomitantly, co-supplementation with calcium plus vitamin D for the prevention of osteoporosis and subsequent fractures is being supported by strong scientific evidence [30].

A complementary approach to manage disease manifestations and improve the quality of life is the consumption of probiotics. Clinical studies on the effect of probiotic supplementation against the onset and management of osteoporosis have shown that probiotic microorganisms can exert favorable effects, by targeting the mechanisms for bone resorption and development, or by modulating immune response and/or the gut microbiome structure (Table 1). More specifically, supplementation with *Bacillus subtilis* C-3102 halted the loss of bone mineral density in postmenopausal women, by modulating bone metabolism and the composition of the gut microbiome [31]. Positive outcomes in bone mineral density of postmenopausal women, were also recorded after supplementation with *Lactobacillus reuteri* ATCCPTA 6475 [32], or with a mixture containing *L. paracasei* DSM 13434, *L. plantarum* DSM 15312, and *L. plantarum* DSM 15313 [33]. On the other hand, co-administration of a probiotic cocktail with 500 mg calcium plus 200 IU vitamin D daily, resulted in the modulation of PTH, pro-inflammatory and bone-turnover markers, but with no significant effect on bone mineral density [34]. The use of fermented dairy products rich in potential probiotic strains in the management of osteoporosis has also been studied. More specifically, Tu et al. conducted a randomized double-blind, placebo-controlled, clinical trial, to investigate the effects of kefir supplemented with calcium bicarbonate in bone metabolism. The kefir-fermented milk-supplemented group showed a significant improvement in bone mineral density, as well as short-term changes in bone-turnover biomarkers, such as reduction in serum β C-terminal telopeptide of type I collagen, reduction in serum osteocalcin, and increase in serum parathyroid hormone [35]. Although, these clinical trials have generated promising results, further studies on the characterization of the mechanism(s) of action of probiotics should be performed, to harvest novel knowledge and design targeted regimes.

### 2.2. Rheumatoid Arthritis

Rheumatoid arthritis is a common chronic systemic autoimmune disease that presents with persistent inflammation in synovial joints and, if left untreated, can lead to articular and extra-articular destruction [20]. The most common symptoms of RA include musculoskeletal pain, edema, stiffness, and decreased functioning, while patients have a serious risk of co-morbid conditions, such as severe infection, respiratory disease, osteoporosis, cardiovascular disease, cancer and psychological problems. The onset of RA is usually between 35 to 60 years. Although, the peak incidence is between ages 50 to 60 years, it is widespread in people over the age of 75, while one third of RA patients are diagnosed at age >60 years [36]. Several pharmacological agents are currently available to alleviate the symptoms of RA, including disease modifying anti-rheumatic drugs, which are widely used to promote remission by slowing the progression of joint destruction. However, several adverse reactions and toxicity have been reported, particularly in elderly patients [37]. The exact pathogenesis of RA remains elusive; however, risk factors include genetic polymorphisms, obesity and lifestyle choices, such as smoking [38]. Additionally, recent studies also show that patients present dysbiosis in the gut and oral microbiomes could be an additional cause for RA onset [39]. However, it is still unclear whether dysbiosis is a driving factor for the initiation of the disease, or a mere consequence.

Probiotics presenting immunomodulatory responses have been tested in the clinic for their ability to limit RA manifestations (Table 1). Indeed, probiotics were shown to alleviate the manifestations of disease and downregulate disease-specific biomarkers, mainly by regulating inflammatory responses and reducing oxidative stress. More specifically, probiotic supplementation was shown to reduce C-reactive protein (CRP) [40] or tumor necrosis factor (TNF)-α and interleukin (IL)-6 levels, as well as oxidative stress [41]. Furthermore, *L. acidophilus, L. casei* and *Bifidobacterium bifidum* resulted in decreased Disease Activity Score (DAS)-28 and overall improvement of systemic inflammation and metabolic profile of the probiotic-treated individuals [42]. On the contrary, probiotic treatment with *L. rhamnosus* GR-1 and *L. reuteri* RC-14, as an adjunct therapy to RA treatments, suppressed systemic inflammation and possibly joint inflammation, however, clinical examination of patients did not show overall clinical improvement [43].

**Table 1:** The effect of probiotic supplementation on age-related bone diseases.

Probiotic Strains	Participants	Age (Years, Mean ± SD)		Sex Ratio (M/F)		Type of Study	Intervention		Duration of Intervention	Key Molecular Findings	Clinical Outcomes	Ref.
		Probiotic Group	Control Group	Probiotic Group	Control Group		Probiotic Group	Control Group				
Primary Osteoporosis												
*Bacillus subtilis* C-3102	61 healthy post-meno-pausal Japanese women	57.5 ± 4.3	57.8 ± 5.4	All female		Randomized, double-blind, placebo-controlled study	3 capsules, 3.4 × 10^9^ CFU, once daily	Capsules containing dextrin	24 weeks	↑ BMD (total hip) ↓ TRACP-5b ↓ uNTx ↑ *Bifidobacterium* ↓ Relative abundance of *Fusobacterium*	↓ Bone resorption	[31]
*L. reuteri* 6475	70 women with low BMD	76.4 ± 1.0	76.3 ± 1.1	All female		Randomized, double-blind, placebo-controlled trial	Stick packs containing 5 × 10^9^ CFU, twice daily	Stick-packs containing maltodextrin powder	12 months	ΝS	↓ Loss of total vBMD and trabecular bone volume fraction	[32]
*L. paracasei* DSM 13434, *L. plantarum* DSM 15312, *L. plantarum* DSM 15313	234 healthy women in the early post-menopausal phase	59.1 ± 3.8	58.1 ± 4.3	All female		Randomized, double-blind, placebo-controlled, multicenter trial	Capsules, 10¹⁰ CFU, once daily	Placebo capsules, content not mentioned	12 months	ΝA	↓ LS-BMD loss	[33]
*L. casei*, *L. acidophilus*, *L. rhamnosus*, *Bifidobacterium* *breve*, *Streptococcus thermophilus*	41 osteopenic post-menopausal women	58.85 ± 0.68	57.29 ± 0.72	All female		Randomized, double-blind, placebo-controlled study	Multispecies capsules, once daily	Capsules containing 500 mg of corn starch	6 months	↓ BALP ↓ Serum CTX ↓ PTH ↓ Serum TNF-α	NS	[34]
Kefir	40 osteoporotic patients	64.08 ± 14.51	67.94 ± 8.37	7/17	7/8	Randomized, double-blind, placebo-controlled study	1600 mg kefir and 1500 mg CaCO_3_ daily	1600 mg unfermented raw milk and 1500 mg CaCO_3_ daily	6 months	↑ Serum PTH ↓ Serum β-CTX ↑ OC	↑ BMD	[35]
*L. casei* Shirota	381 patients with acute distal radius fracture	64.3 ± 4.1	65.1 ± 3.7	93/96	94/96	Randomized, double-blind, placebo-controlled trial	Skimmed milk containing 6 × 10 ^9^ CFU, twice daily	Skimmed milk	6 months	ΝA	↑ Healing process ↓ DASH score ↓ pain VAS ↓ CRPS score	[44]
Rheumatoid Arthritis												
*Bacillus coagulans* GBI-30	45 patients	62.5		9/36		Randomized, double-blind, placebo-controlled trial	Caplets, 2 × 10 ^9^ CFU, once daily	Capsules containing micro-crystalline cellulose	60 days	↓ CRP	Improvement in: patient pain assessment score, pain scale, patient global assessment and self-assessed disability	[40]
*L. acidophilus* La-14, *L. casei* Lc-11, *Lactococcus lactis* Ll-23, *B. lactis* Bl-04, *B. bifidum* Bb-06	42 patients	59	57	3/18	2/19	Randomized, double-blind, placebo-controlled trial	Sachet with freeze-dried bacterial strains, 10^9^ CFU/g of each strain, once daily	Capsules containing maltodextrin	2 months	↓ WBC ↓ TNF-α ↓ IL-6 ↓ NO_x_ ↑ SH ↑ TRAP	ΝS	[41]
*L. acidophilus*, *L. casei, Bifidobacterium* *bifidum*	60 patients	52.2 ± 12.2	50.6 ± 13.1	5/25	4/26	Randomized, double-blind, placebo-controlled trial	Capsules, viable and freeze-dried strains 2 × 10^9^ CFU/g of each strain, once daily	Capsules containing starch	8 weeks	↓ Serum insulin ↓ hs-CRP ↓ HOMA-B ↑ Plasma GSH Improved VAS	Improved DAS-28	[42]
*L. rhamnosus* GR-1, *L. reuteri* RC-14	29 patients	63.8 ± 7.5	59.1 ± 9.1	1/14	1/13	Randomized, double-blind, placebo-controlled trial	Capsules, 2 × 10^9^ CFU, twice daily	Capsules containing inactive ingredients	3 months	Suppressed pro-inflammatory cytokine production	Improvement of HAQ score, No clinical improvement	[43]

↑: Increased; ↓: Decreased; 2-OH/ -OH: Urinary 2-hydroxyestrone to 16a-hydroxyestrone ratio; ACR: American College of Rhematology Criteria; BALP: Bone-specific alkaline phosphatase; BMD: Bone mineral density; β-CTX: β C-terminal telopeptide of type I collagen; CDM: Calcium, vitamin D, and magnesium; CFU: Colony forming units; CRP: C-reactive protein; CRPS: Complex regional pain syndrome; CTX: Collagen type 1 cross-linked C-telopeptide; DAS-28: Disease activity score of 28 joints; DASH: Disabilities of the arm, shoulder and hand; GSH: Total glutathione; HAQ: Health assessment questionnaire; HOMA-B: Homeostatic model assessment-B cell function; hs-CRP: Highly-sensitive C-reactive protein; IL-6: Interleukin 6; LS-BMD: Lumbar spine bone mineral density; NA: Non available; NO_x_: Nitric oxide metabolites; NS: Non-significant; OC: Osteocalcin; PTH: Parathyroid hormone; RA: Rheumatoid arthritis; RCE: Red clover extract; SH: Sulfhydryl groups of protein; TNF-α: Tumor necrosis factor alpha; TRAP: Total radical-trapping antioxidant parameter; TRACP-5b: Tartrate-resistant acid phosphatase isoform 5b; uNTx: Urinary type I collagen cross-linked N-telopeptide; VAS: Visual analogue scale; vBMD: Volumetric bone mineral density; WBC: White blood cell count; WOMAC: Western Ontario and McMaster Universities osteoarthritis index.

## 3. Age-Related Neurodegenerative Disorders and Probiotics

Neurodegenerative disorders constitute a set of pathological conditions originating from progressive dysfunction of synapses, neurons, glial cells, and their networks. They are either hereditary or sporadic conditions resulting in progressive loss of the structure and function of neurons, ultimately leading to neuronal death. They can be broadly classified by their clinical presentations, with Alzheimer’s and Parkinson’s diseases being the most common, as they are observed in one in ten elderly individuals over 65 years old [45]. A primary feature of both diseases is the deposition of physiochemically modified variants of normal proteins in the nervous system, not only in neurons but also in glial cells [46]. The aggregation of these proteins in the brain promotes neuro-inflammation and increased oxidative damage, amplifying the manifestations of disease [47]. More specifically, the aggregates can bind to pattern recognition receptors on microglia and trigger inflammatory signaling pathways, resulting in the secretion of several proinflammatory cytokines, such as TNF-α, interferon (IFN)-γ and IL-1β, -6 and -18, with the aim of neutralizing the toxic insults. When the toxic stimuli are cleared, the microglia begin to secrete anti-inflammatory cytokines such as IL-4, IL-10 and IL-18, a brain-derived neurotrophic factor (BDNF) or nerve growth factor (NGF), aiming to terminate innate immune responses. However, under pathological conditions, the overproduction of proinflammatory cytokines and the decrease in neuroprotective agents can lead to neurodegeneration [48]. Concomitantly, evidence suggests that oxidative stress could play a role in neuronal cell death. More specifically, proteins modified by oxygen reactive species tend to form agglomerates, acting as endogenous inhibitors of proteasome activity. The ubiquitin/proteasome system (UPS) and the autophagy–lysosomal pathway are the major intracellular pathways for protein degradation, under physiological conditions, and thus their inhibition could result in apoptotic or necrotic cell death [49]. Some data also reveal the metabolic profile of neurogenerative diseases, showing that patients present systemic insulin resistance and reduced insulin levels in the brain. Under homeostatic conditions, insulin can stimulate dendritic growth, activation of neuronal stem cells, cell growth and repair [50]. Lastly, recent advancements in microbiome research have contributed to the understanding of the bidirectional gut–microbiome–brain communication, exposing a possible link of gut dysbiosis with neurogenerative disease onset [51].

### 3.1. Alzheimer’s Disease

Alzheimer’s disease (AD) is the most common neurodegenerative disease and the leading cause of dementia in the elderly, accounting for about 60–80% of total cases. Cognitive impairment in at least one cognitive area, memory loss, inability to learn and concentrate, behavioral and psychological disorders are some of the key clinical characteristics of the disease [52]. The pathogenesis of the disease has been attributed to the presence of two types of abnormal structures in the cortical and peripheral regions of the human brain, extracellular aggregates of amyloid β (Aβ) plaques and intracellular neurofibrils of hyperphosphorylated protein τ [53]. AD also presents an inflammatory basis [54] and increased oxidative stress [55], gut dysbiosis [51] and insulin resistance [56]. Experimentally, several therapeutic strategies that aim to inhibit the progression of the disease through the degradation of abnormal protein aggregates have been proposed and tested in vivo, however, their implementation at the clinical level has not been so successful [57]. This fact, combined with the gradual progression of AD suggest that amyloid-beta peptide accumulation may begin 10–20 years before the onset of clinical evidence, necessitating the investigation of new possible intervention strategies to delay the onset of the disease [57].

To date, clinical studies on the effect of probiotics on AD, have mainly focused on the regulation of systemic effects of the disease, with the aim to retard its onset and alleviate related manifestations. Specifically, probiotic administration has been associated with decreased plasma malondialdehyde (MDA) and serum high sensitivity C-reactive protein (hs-CRP) levels [58], as well as elevated total glutathione (GSH) concentration [59]. Both studies recorded improvements in scores in the homeostasis model of assessment of insulin resistance (HOMA-IR) and quantitative insulin sensitivity check (QUICKI) indexes. These molecular changes were accompanied by improvement of the Mini-Mental State Examination (MMSE) scores in the intervention groups. Accordingly, kefir consumption decreased proinflammatory cytokine levels (TNF-α, IL-8, IL12p70, IL-8/IL-10 and IL-12/IL-10), as well as oxidative stress markers (·O2–, H2O2, and ONOO−) and increased NO bioavailability [60]. The direct effects of probiotic consumption to brain function of AD patients, were studied by Hwang et al. [61]. The authors found that supplementation of AD patients with *L. plantarum* C29 led to improved cognitive performance and increased serum brain-derived neurotrophic factor (BDNF). Concomitantly, an increase of the lactobacilli populations in the gut was also recorded [61]. This is the only available study that indicates an improvement in cognitive performance. On the other hand, no effect on either brain activity or biomarkers of disease was recorded after supplementation with *B. breve* A1 [62] or *L. acidophilus, L. casei, L. fermentum*, and *B. bifidum* [63], underpinning the need for case-by-case investigation of species-specific actions and personalized interventions.

### 3.2. Parkinson’s Disease

Parkinson’s disease (PD) is the second most common neurodegenerative condition following AD. The manifestations of the disease include both motor and non-motor symptoms, such as tremors at rest, slowness of movement, muscle rigidity, gastrointestinal disorders and mental conditions such as dementia, depression and apathy [64]. Most cases are of sporadic occurrence; however, risk factors contributing to the manifestation of disease are unknown. On that note, aging is considered as the most important risk factor, as the median age of PD onset is 60 years [65]. The pathogenesis of PD is characterized by neuronal loss in the substantia nigra, causing striatal dopamine deficiency. Apart from dopamine neurons, research indicates the degeneration of noradrenergic, glutamatergic, serotonergic and adenosine neurons, that can partially provide an explanation for the non-motor symptoms of PD. Neuron degeneration is the outcome of intracytoplasmic inclusion bodies, also termed as Lewy bodies, mainly containing aggregates of α-synuclein. The accumulation of these aggregates can damage mitochondrial function, and cause nucleus and microtubule network degeneration [66]. Furthermore, PD shares a strong neuroinflammatory and pro-oxidative profile with AD, contributing to neuronal dysfunction. To date, the mechanism that triggers the aggregation of α-synuclein and formation of Lewis bodies is not quite understood [67]. An interesting new hypothesis supported by animal studies is the production of aggregates in the gut by microbial residents and their transport to the lower brain stem via the vagus nerve [68]. The translation of these findings to the clinical setting could reveal novel therapeutic targets.

Today, clinical studies on the effect of probiotic supplementation on PD have mainly focused on the alleviation of non-motor symptoms of the disease. The majority of patients with PD suffer from gastrointestinal disorders such as constipation, nausea and vomiting, as well as increased intestinal permeability, also known as intestinal leakage, leading to serious complications, such as intestinal pseudo-obstruction and volvulus. Such conditions reflect changes in the intestinal microbiome [69]. Indeed, changes in the abundance of gut bacterial families has been recorded among PD patients. For instance, Scheperjan et al. observed a significant reduction in *Prevotellaceae* in fecal samples of PD patients, compared to the control group, which resulted in gut dysbiosis, while the relative abundance of *Enterobacteriaceae* was positively correlated with the severity of postural instability and gait difficulty in PD patients [70]. Hill-Burns et al. reported a higher abundance of *Lactobacillaceae* in PD patients when compared with controls, in addition to other bacterial families [71]. Using 16S rRNA gene sequencing of microbial DNA found in stool, Hopfner et al. found that *Lactobacillaceae*, *Barnesiellaceae*, and *Enterococcaceae* are more abundant in the gut of PD patients [72]. However, a consensus on microbiome alterations that are linked to the onset and progression of PD has yet to be established. Probiotic interventions aim mainly at enhancing the quality of life of the individuals with PD, and thus several placebo-controlled trials have been conducted, showing beneficial effect of probiotic supplements on symptoms of gastrointestinal dysfunction. For example, multi-species supplementation containing lactobacilli, *Bifidobacterium* and *Streptococcus* strains to elders with PD, resulted in better bowel habits [73], alleviated abdominal pain and bloating [74], improved stool consistency and quality of life related to constipation [75], as well as increased bowel opening frequency and gut transit time [76]. Accordingly, insulin homeostasis and markers of insulin metabolism, such as HOMA-IR and QUICKI have been significantly improved in PD patients, after consumption of bacterial consortia including probiotic strains. These molecular changes were accompanied by better scorings of the Movement Disorder Society-Sponsored Revision of the Unified Parkinson’s Disease Rating Scale (MDS-UPDRS) [77] (Table 2).

## 4. Cancer in the Elderly and Probiotics

According to recent epidemiological data colorectal cancer is the fourth most diagnosed cancer and the third deadliest malignancy in the world [80]. The onset of colorectal cancer is influenced by both genetic (hereditary form) or environmental factors (sporadic occurrence). Most patients with sporadic cancer are over 50 years old, while 75% of patients with rectal cancer and 80% of patients with colon cancer are over 60 years old at the time of diagnosis [81]. The biological basis of disease includes genomic instability, impairment in the DNA damage response, as well as epigenetic changes [82]. Furthermore, local inflammatory responses are heightened, leading to loss of gut barrier integrity and systemic inflammation [83]. The weakening of the gut barrier can result in leakage of the intestinal content, triggering sub-epithelial immune cell populations and leading to exacerbated inflammatory responses [84]. Interestingly, emerging data suggest that microbial dysbiosis in the human intestine and the proliferation of tumorigenic species may play an important role in colon carcinogenesis. *Fusobacterium nucleatum* is one of the strains that has been implicated as a stimulator of colon oncogenesis. This strain inhabits the human oral mucosa and can translocate to distant sites via blood circulation. When in the human colon, it can colonize epithelial cells and trigger pro-inflammatory responses, while also interfering with cellular pathways, increasing cell proliferation and chemoresistance [85]. Colon cancer is mainly managed by surgery, that is usually preceded by mechanical bowel preparation (MBP) [86]. Although of vital importance, surgery can result in surgical trauma, and serious postoperative infections [87]. These interventions can also have taxing effects on the microbiome, resulting in dysbiosis and heightened gastrointestinal side effects. More specifically, reduced populations of bifidobacteria, *Clostridium coccoides*, *C. leptum*, *Enterobacteriaceae* and *Lactobacillus* have been recorded, postoperatively [88]. Accordingly, Drago et al. observed significant changes in the composition of the intestinal microflora and, in particular, reduced abundance of *Lactobacillaceae* and increased abundance of *Enterobacteriaceae* and *Streptococcaceae*, which remained for up to 30 days, following surgical resection [89]. Since non-pathogenic bacteria make up the vast majority of the intestinal microbiome, MBP ultimately appears to be directed primarily against populations that are beneficial to the organism, allowing pathogens, such as *Escherichia* and *Staphylococcus* to thrive. Antibiotic prophylaxis, either orally or systemically, is considered a valuable way of treating and preventing postoperative infections, however their broad-spectrum action makes them less suitable, as in addition to pathogens, they destroy beneficial bacteria and reduce the health benefits of diversity and abundance of intestinal microflora [87]. For instance, Young et al. observed that administration of amoxicillin-clavulanic acid for 10 days almost eliminated *Bifidobacterium* spp., while the abundance of the genus was not restored even 20 days later [90]. Chemotherapy plays an equally important role in the treatment of colon cancer; however, the extensive cytotoxicity of chemotherapeutic drugs can disrupt the gut microflora and damage the gastrointestinal mucosa (gastrointestinal mucositis). This can not only limit the effectiveness of chemotherapy but can also severely affect the quality of life of the patient [91,92]. It is important to note that the use of common chemotherapeutic drugs can lead to intestinal dysbiosis, which is characterized by a reduction in microbes, such as *Bifidobacterium* and *Lactobacillus* and an increase in opportunistic pathogens [93]. In general, all the mentioned therapeutic interventions inevitably cause intestinal dysbiosis, disrupt the intestinal barrier, destroy the normal gut function, and may favor proliferation of pathogenic microorganisms [87]. Their clinical impact includes surgical site infections and gastrointestinal symptoms, such as diarrhea and constipation, prolonging the hospitalization time and having a direct impact on the patient′s recovery [94]. Probiotic supplementation as an adjunct method to manage these unwanted side effects have been based on the anti-inflammatory activity of strains, as well as on their ability to modulate the gut microbial composition and exert antimicrobial effects.

Clinical studies show that perioperative (postoperative and/or preoperative) administration of probiotics may accelerate the recovery of the intestinal microbial composition and prevent possible gastrointestinal side effects. Indeed, adjunct probiotic supplementation was shown to result in gut microbiota composition alterations, such as an increase in abundance of *Bifidobacterium* [95,96] and a decrease in *Escherichia* counts [95]. Importantly, surgical site infection clearance [97,98] and faster restoration of normal gut and bowel function [99], decreased incidence of diarrhea [100], elevated incidence of bacterial translocation and decreased intestinal permeability have also been reported [98] (Table 3). Moreover, colorectal cancer patients receiving chemotherapy in combination with probiotic supplements showed improved quality of life and a significant improvement in gastrointestinal function, with a reduction in the incidence of diarrhea and the onset of enterocolitis [101,102]. Furthermore, the administration of probiotics to patients with colon cancer before and/or after their planned resection showed significant changes in their immune profile. More specifically, Zaharuddin et al. evaluated changes in a wide range of circulating inflammatory cytokines and reported reduced levels of the proinflammatory cytokines, TNF-α, IL-6, IL-10, IL-12, IL-17A, IL-17C and IL-22 [103]. In another study, patients who received probiotics showed lower levels of serum IL-6 and CRP, while simultaneously presenting higher levels of IgG and sIgA, after intervention. These findings indicate that probiotic consumption limited systemic stress markers, while also improving local immunity. Consequently, the abundance of enteropathogens was decreased and markers of intestinal overgrowth, such as D-lactic acid and endotoxin were also lower in the treated group [104]. In this context, supplementation with a mixture containing lactobacilli and bifidobacteria also induced changes in the gut microbiome composition, however, these alterations were not reflected in the clinical phenotype of the patients [105]. Accordingly, perioperative supplementation with *B. bifidum* did not manage to limit the occurrence of infections, post-surgery [106].

Probiotics have also been tested as an alternative to manage chemotherapy, immunotherapy and radiation side effects, in patients with extraintestinal tumors. Radiation therapy is an effective treatment that kills cancer cells using high-energy rays or particles. Today it remains an integral part of cancer treatment with approximately 50% of all cancer patients, including those with prostate cancer, receiving radiotherapy [107]. Radiotherapy plays a major role for the management of patients with rectal cancer and is given either in the neoadjuvant setting with or without chemotherapy, or in the adjuvant setting after rectal cancer surgery. Gastrointestinal side effects are quite common, and it is estimated that over 70% will develop acute symptoms, with the most prominent of these being diarrhea, a debilitating condition resulting in dehydration, electrolyte imbalance, malnutrition, fluid depletion and extended hospitalization [108]. The main cause behind this side effect appears to be located in the malabsorption of lactose and bile acids, in the changes of the intestinal flora and intestinal motility resulting in impaired secretion, absorption and immune function of the digestive system [109]. Recent data suggest that the use of probiotics may be effective in preventing radiation-induced diarrhea in high-risk patients, who receive radiation to the lower abdomen and pelvis. Patients with pelvic cancer (including prostate cancer) receiving radiotherapy, with or without chemotherapy, were treated with probiotics throughout treatment and the results showed reduction in the incidence of all grade diarrhea [110]. Diarrhea is a common side effect of chemotherapy regimens that include fluorouracil and irinotecan, with an incidence of up to 50–80% of patients, a large proportion of whom can develop severe symptoms. In this context, patients diagnosed with lung cancer were given probiotics in combination with chemotherapy, and the results showed relief from diarrhea and reduction in the systemic inflammatory responses [111].

**Table 3:** The effect of probiotic supplementation on the management of cancer in the elderly.

Probiotic Strains	Participants	Age (Years, Mean ± SD)		Sex Ratio (M/F)		Type of Study	Intervention		Duration of Intervention	Key Molecular Findings	Clinical Outcomes	Ref.
		Probiotic Group	Control Group	Probiotic Group	Control Group		Probiotic Group	Control Group				
Colorectal Cancer												
*E. faecalis* T110, *Clostridium butyricum* TO-A, *Bacillus mesentericus* TO-A	156 CRC patients scheduled for surgery	68.0 ± 13.8	69.1 ± 11.3	47/28	44/37	Rando-mized clinical trial	Multispecies tablets, 6 times daily	No placebo	3–15 days before surgery	↑ Adenosine triphosphate ↑ *Bifidobacterium* abundance	↓ Superficial incisional SSIs ↑ Immune responses	[96]
*L. acidophilus* LA-5, *L. plantarum*, *B. lactis* BB-12, *S. boulardii*	164 CRC patients scheduled for elective, open, colonic resection	65.9 ± 11.5	66.4 ± 11.9	57/27	58/22	Rando-mized, double-blind, placebo-controlled trial	Multispecies capsules, twice daily	Placebo capsules containing powdered glucose polymer	16 days; 1 day before major colorectal surgery and 15 days post-operatively	Expression of SOCS3 → positively related with expression of TNF-α and circulating IL-6	↓ Major postoperative complications	[97]
*L. plantarum*, *L. acidophilus*, *B. longum*	156 CRC patients scheduled for radical colectomy	66.06 ± 11.02	62.28 ± 12.41	38/37	40/35	Randomized, double-blind, placebo-controlled trial	Multispecies capsules, total daily dose of 2.6 × 10^14^ CFU	Placebo capsules containing maltodextrin	16 days; 6 days before and 10 days after surgery	↓ Postoperative serum zonulin Inhibition of the p38 MAPK signaling pathway	↓ Bacterial translocation ↓ Intestinal permeability ↓ Pyrexia ↓ Duration of antibiotic therapy	[98]
*L. acidophilus* BCMC^TM^12130, *L. casei* BCMC^TM^12313, *L. lactis* BCMC^TM^12451, *B. bifidum* BCMC^TM^02290, *B. longum* BCMC^TM^02120, *B. infantis* BCMC^TM^02129	40 CRC patients scheduled for surgery	64.3 ± 14.5	68.4 ± 11.9	11/9	13/7	Rando-mized, double-blind, placebo-con-trolled trial	Sachets, 3 × 10^10^ CFU, twice daily	Placebo capsules, content not mentioned	7 days before surgery	ΝA	Faster return of normal gut function Faster recovery Shorter duration of hospital stay	[99]
*B. longum*, *L. acidophilus*, and *E. faecalis*	60 CRC patients scheduled for confined colorectal cancer resection operation	63.90 ± 12.25	62.17 ± 11.06	15/15	12/18	Rando-mized, double-blind, placebo-con-trolled trial	Probiotic powder, 10^7^ CFU/g of each strain, 2 g daily	Placebo powder containing maltodextrin and sucrose	12 days; 5 days before and 7 days after CRC resection operation	NS	↓ Incidence of diarrhea Faster recovery of bowel function	[100]
*B. breve* HA-129, *B. bifidum* HA-132, *B. longum* HA-135, *L. rhamnosus* HA-111, *L. acidophilus* HA-122, *L. casei* HA-108, *L. plantarum* HA-119, *S. thermopilus* HA-110, *L. brevis* HA-112, *B. infantis* HA-116	46 CRC patients starting new line of chemotherapy	62	64	14/9	12/11	Rando-mized, double blind, placebo con-trolled pilot study	Capsules, 10^10^ CFU, 3 times daily	Placebo capsules, only inactive ingredients	12 weeks	NA	↓ Incidence of severe diarrhea of grade 3 or 4 ↓ Overall incidence of diarrhea ↓ Incidence of enterocolitis	[101]
*B. infantis*, *L. acidophilus*, *E. faecalis*, *B. cereus*	100 CRC patients undergoing chemo-therapy	62.1 ± 10.9	60.1 ± 9.9	35/15	33/17	Rando-mized clinical trial	4 tablets (con-centration not reported), 3 times daily	No placebo	4 weeks	NS	Alleviated functional constipation during chemotherapy	[102]
*L. acidophilus* BCMC^®^ 12130, *L. lactis* BCMC^®^ 12451, *L. casei* BCMC^®^ 12313, *B. longum* BCMC^®^ 02120, *B. bifidum* BCMC^®^ 02290, *B. infantis* BCMC^®^ 02129	52 CRC patients scheduled for surgery	67.33 ± 9.4	66.5 ± 8.5	19/8	15/10	Rando-mized, double-blind, placebo-controlled trial	Mixture, 3 × 10^10^ CFU, twice daily	Placebo capsules -content not mentioned	6 months starting 4 weeks after surgery	↓ TNF-α ↓ IL-6 ↓ IL-10 ↓ IL-12 ↓ IL-17A ↓ IL-17C ↓ IL-22	Safety of probiotic consumption	[103]
*B. longum*, *L. acidophilus*, *E. faecalis*	60 CRC patients scheduled for radical colorectal resection	67.5	61.5	10/20	14/16	Randomized, double-blind, placebo-controlled trial	3 capsules, 10^8^ CFU/g, 3 times daily	Placebo capsules containing malto-dextrin	3 days before surgery	↑ *Bifidobacterium* ↑ *Escherichia* ↓ Endotoxins ↓ D-lactic acids ↓ IL-6 ↓ CRP ↑ IgG ↑ sIgA	↓ Occurrence of infectious complications	[104]
*B. animalis* subsp. *lactis* HY8002, *L. casei* HY278, *L. plantarum* HY7712	60 CRC patients scheduled for anterior resection	60.10	61.03	19/10	13/18	Randomized, double-blind, multicenter, exploratory placebo-controlled trial	Probiotic powder, twice daily	Placebo powder of prebiotics and sugars	4 weeks, starting at one week before surgery	Compositional changes in gut microbiota ↓ Serum zonulin	NS	[105]
*B. bifidum*	294 CRC patients scheduled for elective colon cancer operation	67 ± 9	66 ± 12	49/51	51/44	Prospective randomized trial	3 tablets, 10^10^ CFU, 3 times daily	No placebo	17 days total; 7 days before surgery, 10 days after surgery	NS	NS	[106]
Pelvic Cancer												
*L. acidophilus* LAC-361, *B. longum* BB536	229 pelvic cancer patients receiving radio-therapy treatments	61.7	60.6	97/43	56/33	Randomized, double blind, placebo controlled study	Capsule, 1,3 × 10^9^ CFU, twice daily (standard dose) or 10 × 10^9^ CFU, 3 times daily (high dose)	Placebo tablets -content not mentioned	During the radiation therapy treatments	NS	↓ Radiation induced grade 2–3-4 diarrhea	[110]
Lung Cancer												
*Clostridium butyricum*	41 patients with lung cancer undergoing chemo-therapy	57 ± 8.75	54 ± 8.35	15/5	15/6	Randomized, double blind, placebo controlled study	3 tablets (420 mg/tablet), 3 times daily	Placebo tablets -content not mentioned	3 weeks	↓ NLR ↓ PLR ↑ LMR at week 3 ↑ *Clostridium* and *Lactobacillus* genera	↓ Chemotherapy-induced diarrhea Alleviated inflammatory response Maintained gut homeostasis	[111]

↑: Increased; ↓: Decreased; BT: Bacterial translocation; CFU: Colony-forming unit; CRC: Colorectal cancer; CRP: C-reactive protein; EPA: Eicosapentaenoic acid; IgG: Immunoglobulin G; IL: Interleukin; LMR: Lymphocyte/monocyte ratio; NA: Not available; NLR: Neutrophil/lymphocyte ratio; NS: Non-significant; p38MAPK: p38 mitogen-activated protein kinase; PLR: Platelet/lymphocyte ratio; QOL: Quality of life; sIgA: Secretory immunoglobulin A; SSIs: Surgical site infections; SOCS3: Suppressor of cytokine signaling 3; TNF-α: Tumor necrosis factor alpha.

## 5. Probiotics and Aging; Pitfalls and Future Perspectives

### 5.1. Other Diseases of Aging

The physiological aging process is characterized by a progressive loss of resilience and homeostasis. Cell senescence and the exhaustion of the regenerative mechanisms result in loss of tissue functionality, thus providing fertile ground for the onset of multimorbidity. Apart from cancer, neurodegenerative and musculoskeletal disorders; cardiovascular and metabolic disease also present with high frequencies in this demographic [3], as aging is a major risk factor for chronic inflammatory diseases, such as diabetes and atherosclerosis. In this context, cell senescence and telomere shortening in cardiac cells lead to the progressive degeneration of aortic valves and vascular cells, increasing risk for the incidence of stroke and cardiac arrest [2]. Obesity and aging are the major risk factors for the development of type 2 diabetes mellitus (T2DM) [4]. Elevated glucose and lipid levels can, in turn, accelerate cellular senescence locally (adipose tissue) and systemically [112]. Subsequently, T2DM complications can lead to kidney dysfunction, hepatic steatosis and promote the onset of other endocrine conditions [4]. Interestingly, the gut microbiota can present differences between prediabetic and healthy individuals, as shown during the integrative human microbiome project (iHMP). Indeed, it was found that insulin-resistant participants exhibited a specific metabolic profile, delayed inflammatory responses, and altered gut microbiome structure compared to insulin-sensitive participants. Importantly, this multilevel approach was efficient in pinpointing disease states prior to clinical manifestations [113]. T2DM and its co-morbid conditions can seriously affect the quality of life of elders and increase their dependency, however a plethora of efficient medication regimes are available to patients. In this context, novel studies suggest tackling the root causes of disease (the shared underlying biological processes) rather than disease-specific approaches, with the ultimate goal of retarding their onset. More specifically, a systematic metanalysis on the effect of probiotic supplementation in markers of metabolic disease, showed that the participants presented decreased insulin resistance and lower concentration of plasma glucose, suggesting that probiotics could act complementary to T2DM medication [114]. It is important to note however, that this meta-analysis used heterogenic studies to draw these conclusions, and thus no specific probiotic regimen could be identified as the most beneficial. Accordingly, in another meta-analysis, the effect of probiotic supplementation and fermented food consumption on individuals with increased risk of cardiovascular disease was investigated. It was found that probiotic consumption led to improved health outcomes, namely the reduction of blood pressure, serum cholesterol, triglycerides, and glucose, as well as a decrease of low-density lipoprotein (LDL) levels [115]. Subgroup analysis showed that the positive outcomes were significantly correlated with higher probiotic dose and duration of treatments and the use of fermented products rather than probiotic supplements [115]. The efficacy and safety of these interventions are currently under investigation and no specific guidelines have been established for their use.

### 5.2. Deciphering the Mechanisms of Probiotic Action in Aging

Preclinical studies on ageing are performed in vertebrate and invertebrate models; common mice (*Mus musculus*) and rat (*Rattus norvegicus domestica*) strains, as well as the fruit fly (*Drosophilla melanogaster*), roundworm (*Caenorhabditis elegans*) or zebrafish (*Danio rerio*) and turquoise killifish (*Nothobranchius furzeri*) [116]. Accordingly, species that present exceptionally high longevity, such as naked mole rats, Greenland sharks, whales, hydra and jellyfish are used to decipher the mechanisms that may be involved in this phenomenon [117]. Although the use of short-lived animals is advantageous for laboratory research, their employment in translational aging research presents several drawbacks, as they rarely present age-related diseases. On the other hand, the use of primates can provide better insights into the pathophysiology of these diseases, constituting a more precise model for the study of the effect of novel compounds on longevity and disease onset [118]. Despite all these data, rodents are the most recruited laboratory animal in aging studies. Several mutants have been established for the study of the nine hallmarks of aging and of the effect of senolytic compounds [117], while there are available models that can recapitulate specific age-related disease phenotypes [119]. Probiotic research in aged mice have shown that the consumption of beneficial bacterial strains could improve cognitive and gastrointestinal function, stimulate immune responses, and alleviate hypertension [120]. Mechanistic insights into these data have shown that probiotic strains can exert strain specific results via the modulation of pathways involved in inflammatory and/or insulin signaling and oxidative stress response [120].

The presented preclinical studies have inherent drawbacks, as previously described, while clinical trials may be prone to several pitfalls. More specifically, it is not uncommon for the studies to be underpowered or for the clinical outcomes not being meticulously recorded. In this context, in many of the aforementioned studies, changes in putative biomarkers of inflammation, oxidative stress or other disease-specific markers were recorded, however the clinical outcomes were not described. Furthermore, many clinical studies rely on questionnaires, as primary outcomes for changes in disease burden or in quality of life, which can be prone to bias [121]. Another significant issue is that the probiotic action of administered strains is rarely being studied at length, prior to their introduction to the clinic, while most clinical studies use probiotic cocktails and no single strains. As a result, it is unclear whether the effects are induced by a single microorganism or a combination of probiotics. Additionally, population dynamics are not taken into consideration, and thus possible inhibitory interactions could occur between the strains [16]. In this context, the safety profile of these interventions could be questioned, especially in cocktails containing *Enterococcus* strains, or other potential pathogenic bacteria [122]. Finally, the inclusion of fermented products in these studies may lead to several inconsistencies, as the beneficial effects could be derived from the food microbiome, bacterial metabolites and/or other bioactive compounds present in the matrix. Thus, well-structured studies that present high analytical rigor are necessary to derive conclusions about probiotic action on the aged host and to promote the application in the clinic.

### 5.3. Safety of Probiotic Consumption in the Elderly

Probiotics have been consumed, unknowingly, by humans since the invention of fermentation techniques, as adjunct starter cultures throughout the world. These products have been intuitively used for their health-promoting properties, long before the description of probiotic microorganisms. Today, probiotics, mainly lactobacilli and bifidobacteria, are available in the form of fermented products or as supplements. Most of these strains possess the generally regarded as safe (GRAS) status, awarded by the U.S. Food and Drug Administration (FDA), as they do not pose a threat to the wellbeing of the consumer. This status dictates that the strains do not present hemolytic activity or carry virulence and transferable antibiotic resistance genes [123]. In the numerous clinical studies that have been conducted in healthy adults, no serious adverse effects were recorded. However, in the aftermath of the PROPATRIA study, caution has been raised for the consumption of probiotics by ill or frail individuals. In the particular study, higher morbidity was recorded in patients with severe acute pancreatitis that received probiotics parenterally, however thorough investigation by regulatory studies did not show a causative relationship between the intervention and the outcomes [124]. The elderly could present higher risk for lactobacillus bacteremia, small intestinal bacterial overgrowth (SIBO) and other systematic effects that could result from the accumulation of D-lactic acid after probiotic consumption [125]. On the contrary, recent metanalyses show that probiotic supplementation does not induce such adverse effects [126,127,128]. It should be noted however, that the documentation of safety profile and adverse effects of probiotic consumption is usually inadequately performed in the available studies. Thus, clinical studies with higher analytical rigor are needed in order to establish a consensus on the safety of probiotic strains.

Amid safety concerns, an alternative to the consumption of viable strains is the use of metabolites (postbiotics) or of heat-killed/inactivated bacteria (parabiotics) [129]. Postbiotics are complex mixtures of fermentation byproducts that can include a plethora of proteinic, peptide, lipid and polysaccharide bioactive compounds. Accordingly, heat treatments of bacteria result in the rupture of cell walls and thus the release of cytoplasmic content and of molecules attached to the cell wall and membrane, such as pili, lipoteichoic acids and peptidoglycans [130]. The targeted study of these biomolecules and the characterization of their biological activity presents many advantages, and most importantly their use in pure form [131]. The study of safety profiles of postbiotics and parabiotics is easier, and no adverse effects related to the translocation and proliferation of ingested viable strains can be induced. Concerning the efficacy of these interactions, clinical studies on the effect of tyndallized bacteria and postbiotics have found a positive effect against gastrointestinal and extra-intestinal diseases in the elderly, mainly by priming immune responses [132,133].

### 5.4. Refining Probiotic Research in the Elderly

Aging is highly personalized process, and thus the genetic, metabolic and microbial signature of advanced age could differ between individuals. In a novel study, Ahadi et al. conducted longitudinal and deep multi-omic profiling of clinical samples from individuals aged 25–75 to investigate person-specific signatures of aging. More specifically, the participants were categorized based on the pathways enriched during aging; some participants showed higher expression of immune-related pathways, and others, alterations in pathways linked to cardiac, liver or kidney dysfunction [134]. These results may provide a basis for the differential pace of aging recorded in individuals, as well as the onset of (multi-)morbidity with age. The genetic component of age-related diseases was examined in a recent study, where it was found that diseases that present with late onset in the population, share a common genetic basis. Indeed, there is significant overlap between diseases in terms of loci implicated in longevity [135]. The role of the gut microbiome in ageing is currently a hot topic of study. Microbial residents of the gut co-evolve with the host throughout life [136]. The structure of the gut microbiome is stabilized at around three years of age; during adulthood the composition and function of these communities remain relatively stable, as they can be influenced by a plethora of genetic and environmental factors. Disease-specific microbial signatures during adulthood have been proposed by several studies [10], however in the case of aging, the gut microbiome undergoes tremendous changes leading to dysbiosis. A shared characteristic of the aging gut microbiome is that the diversity of the microbiome falls dramatically, presenting high interindividual variability. This new unstable composition favors the establishment and proliferation of pathobionts, such as Proteobacteria [137]. These changes can trigger local and systemic inflammation, while also contributing to the weakening of the gut barrier integrity [138]. More specifically, the population shifts result in changes in the metabolic profile of the gut microbiome. For instance, decrease in the populations of short-chain fatty acid (SCFA) producers, such as *Akkermansia muciniphila*, results in decreased production of acetate, butyrate and propionate, which display anti-inflammatory activity and preserve the function of the gut mucosa [139]. Ιt is important to note however, that these changes may not be exclusively associated to aging, but also to environmental factors, use of medications (for co-morbid diseases/antibiotics), as well as malnutrition [140].

Τhe gut microbiome can be easily manipulated extrinsically; however, the ability of probiotics to alter its structure and function is debatable. Studies on healthy adults have shown that the gut microbiome presents an individual-specific resistance to the colonization of probiotics [141,142], that may be decreased after antibiotic treatments [142]. Nevertheless, their ability to rehabilitate the structure and function of the gut microbiome is limited and may even have adverse effects by slowing down the full repopulation of the gut [142]. In this light, the fact that the aged microbiome presents a decreased diversity could indicate that probiotic supplementation could more readily modify the gut microbiome. Indeed, a systematic metanalysis showed that probiotic consumption can affect the overall structure of the gut microbiome with varying degrees of success that can be attributed to interindividual differences [143]. Some probiotic strains, however, may not be successful in altering the composition of the gut microbiome, but rather exert their effects on its transcriptomic and metabolic profile. For example, *L. rhamnosus* GG consumption influenced the global transcriptome profile of the gut microbes, increasing the expression of adhesion and motility proteins, while it also impacted pathways related to glycolysis. These changes were correlated with specific gut microbial species [144]. Under the light of these evidence, probiotic supplementation in the elderly should be catered towards the individual, taking into consideration the genomic, metabolic and microbial profile. Multi-omic analysis of the host can support the profiling of responses after probiotic supplementation and elevate the efficacy of interventions. However, these holistic approaches are still rare in the elderly.

Apart from host-related factors, probiotics act in a highly strain-specific manner. Indeed, whole genome sequencing and comparative genomics have revealed a wide range of heterogeneity between bacteria belonging to the former *Lactobacillus* genus, that led to its division into 25 new genera [145]. Only a subgroup of lactobacilli can be termed as probiotics, that present confirmed health-promoting properties, such as anti-inflammatory, immunomodulatory [146], antibacterial, antibiofilm [147] actions, contrary to common misconceptions. The increased use of omics platforms to study the biology of probiotic microorganisms at multiple levels, as well as host response can reveal their mechanisms of action and provide a basis for targeted interventions [148]. More specifically, profiling the metabolic capacity of the strains and the production and secretion of bioactive compounds has shed light on their biological potential. Furthermore, insights into the conditions that promote their production, could streamline their application in the food and pharmaceutical industries. Indeed, studies have shown that probiotic strains can produce differential compounds, when cultured in laboratory media or food matrices [149] or in association with the host [150]. Undoubtedly, the integration of systems biology in probiotic research has unraveled the great complexity of their biological properties, also providing an explanation for contradicting clinical data and inconsistencies of clinical outcomes in individuals.

## 6. Conclusions

As the worldwide population is ageing rapidly, the need for expanding citizen health span is coming to the forefront. Age is considered an important risk factor for the development of debilitating disease that can increase the dependency of individuals and negatively affect their quality of life. The biological mechanisms of aging are starting to be revealed, and novel approaches, for more efficient management of multimorbidity have been developed. Probiotics that can modulate the root causes of aging, especially inflammation, oxidative stress and cell senescence could comprise useful tools in this direction. Despite the available literature on the beneficial effects of probiotic consumption on age-related diseases, no consensus has been reached for their use in clinical practice. This phenomenon could be attributed to the absence of meticulous characterization of the biology and mechanisms of action of probiotic strains that can enhance the lack of translatability of preclinical studies. Furthermore, current clinical studies present analytical drawbacks that can weaken their arguments and conclusions. With the dawn of the multi-omics era, the use of high-throughput platforms to understand the complex host–microbiome–probiotic interactions, could enhance the efficacy and safety of probiotic consumption in the elderly. Conclusively, clinical studies with greater rigor and proper measurement of outcomes to evaluate and systematically classify the holistic effects of probiotic consumption, are required in order to design personalized approaches for the management of age-related disease.

## Figures and Tables

**Table 2:** The effect of probiotic supplementation on age-related neurodegenerative disorders.

Probiotic Strains	Participants	Age (Years, Mean ± SD)		Sex Ratio (M/F)		Type of Study	Intervention		Duration	Key Molecular Findings	Clinical Outcomes	Ref.
		Probiotic Group	Control Group	Probiotic Group	Control Group		Probiotic Group	Control Group				
Alzheimer’s Disease												
*L. acidophilus*, *L. casei*, *B. bifidum*, *L. fermentum*	60 patients	77.67 ± 2.62	82.00 ± 1.69	6/24	6/24	Rando-mized, double-blind, and controlled clinical trial	Probiotic milk, 200 mL/day (2 × 10^9^ CFU/g of each strain)	Milk, 200 mL/day	12 weeks	↓ hs-CRP ↓ HOMH-IR ↓ HOMA-B ↑ QUICKI ↓ TG level ↓ VLDL ↓ MDA	Improvement in MMSE score	[58]
*L. acidophilus*, *B. bifidum*, *B. longum*	79 patients	76.2 ± 8.1	78.5 ± 8.0	NA	NA	Randomized, double-blind, controlled clinical study	2 × 10^9^ CFU of each strain plus selenium (200 mg/day), once daily	Placebo (packaging not reported)	12 weeks	Probiotic plus selenium intake: ↓ hs-CRP ↓ HOMA-IR ↓ FPG ↑ TAC levels ↑ GSH levels ↓ serum insulin ↓ serum TG ↑ QUICKI (compared with only selenium and placebo)	Probiotic plus selenium intake: Improvement in MMSE score	[59]
*Acetobacter aceti*, *L. delbrueckii delbrueckii*, *L. fermentum*, *L. fructivorans, Enterococcus faecium, Leuconostoc* spp., *L. kefiranofaciens, Candida famata*, *C. krusei*	13 patients	78.5 ± 7	-	2/11	-	Uncontrolled clinical trial	Pasteurized milk with 4% kefir grains	-	90 days	↓ TNF-α ↓ IL-8 ↓ IL12p70 ↓ IL-8/IL-10 ↓ IL-12/IL-10 ↓ serum levels of O_2_−, H_2_O_2_, and ONOO−/OH− ↑ serum NO	↑ MMSE score	[60]
*L. plantarum* C29	100 individuals diagnosed with MCI	68.0 ± 5.12	69.2 ± 7.00	20/30	14/36	Rando-mized, double-blind, placebo-controlled clinical trial	DW2009 capsules, 800 mg/day (1.25 × 10^10^ CFU/g)	Placebo capsules containing cellulose	12 weeks	↑ Lactobacilli population	Improve cognitive performance ↑ Sserum BDNF	[61]
*B. breve* A1	19 elderly patients with MCI	82.5 ± 5.3	-	1/18	-	Open-label, single arm study	Capsules, >1 × 10^10^ CFU, twice daily	-	24 weeks	ΝA	↑ MMSE score Improved POMS2 and GSRS scores	[62]
*L. acidophilus*, *L. casei*, *L. fermentum*, *B. bifidum*	48 patients	79.70 ± 1.72	80.57 ± 1.79	7/18	10/13	Rando-mized, double-blind, placebo-controlled clinical trial	Capsules, 3 × 10^9^ CFU, once daily	Placebo capsules containing malto-dextrin	12 weeks	NS	NS	[63]
*B. breve* A1	121 individuals with subjective memory complaints	61.5 ± 6.83	61.6 ± 6.37	30/31	30/30	Rando-mized, double-blind, placebo-controlled trial	Capsules, > 2 × 10^10^ CFU, twice daily	Placebo capsules containing corn starch	12 weeks	NS	NS	[78]
*B. breve* A1	80 healthy older adults with MCI	61.3 ± 7.7	60.9 ± 6.9	19/21	20/20	Randomized, double-blind, placebo-controlled trial	Capsules, 2 × 10^10^ CFU, once daily	Placebo capsules containing maize starch	16 weeks	ΝA	Improvement of cognitive function ↑ RBANS score ↑ JMCIS score	[79]
Parkinson’s Disease												
*Streptococcus salivarius* subsp *thermophilus*, *E. faecium,* *L. rhamnosus* GG, *L. acidophilus, L. plantarum*, *L. paracasei,* *L. delbrueckii* subsp *bulgaricus, B. breve, B. animalis* subsp *lactis*	120 patients	71.8 ± 7.7	69.5 ± 10.3	41/39	24/16	Randomized, parallel group, double-blind, placebo-controlled study	Fermented milk, 250 × 10^9^ CFU, daily	Pasteurized, fermented, fiber-free milk	4 weeks	NA	Improved bowel habits ↑ CBMs	[73]
*L. acidophilus*, *B. infantis*	40 patients	69.80 ± 5.64	75.65 ± 9.66	10/10	7/13	Randomized, parallel group study	60 mg, twice daily	Trimebutine 200 mg 3× day	12 weeks	NA	↓ Abdominal pain ↓ Bloating	[74]
*L. acidophilus*, *L. reuteri*, *L. gasseri*, *L. rhamnosus*, *B. bifidum*, *B. longum, Enterococcus faecalis, E. faecium*	72 patients	70.9 ± 6.6	68.6 ± 6.7	20/14	28/10	Randomized, single-center, double-blind, placebo-controlled study	Capsules, 10^9^ CFU, once daily	Placebo capsules containing an inactive substance	4 weeks	NS (fecal calprotectin levels)	↑ SBM Improved stool consistency and QOL related to constipation	[75]
*L. acidophilus* BCMC^®^ 12130, *L. casei* BCMC^®^ 12313, *L. lactis* BCMC^®^ 12451, *B. infantis* BCMC^®^ 02129, *B. longum* BCMC^®^ 02120	55 patients	69.0	70.5	16/9	17/10	Rando-mized, double-blind, placebo-controlled, study	Capsules, 3 × 10^10^ CFU, twice daily	Granulated milk containing lactose	8 weeks	NA	↑ BOF ↓ GTT	[76]
*L. acidophilus*, *B. bifidum*, *L. reuteri,* *L. fermentum*	60 patients	68.2 ± 7.8	67.7 ± 10.2	ΝA	ΝA	Rando-mized, double-blinded, placebo-controlled trial	Capsules, 8 × 10^9^ CFU/g, once daily	Placebo capsules	12 weeks	↓ Serum insulin, ↓ HOMA-IR, ↓ QUICKI, ↓ hs-CRP, ↓ MDA, ↑ GSH ↓ TG ↓ VLDL-cholesterol levels	↓ MDS-UPDRS	[77]

↑: Increased; ↓: Decreased; AD: Alzheimer’s disease; BDNF: Brain-derived neurotrophic factor; BMs: Bowel movements; BOF: Bowel opening frequency; CBMs: Complete bowel movements; CFU: Colony-forming unit; FPG: Fasting plasma glucose; GI: Gastrointestinal; GTT: Gut transit time; GSH: Total glutathione; GSRS: Gastrointestinal symptom rating scale; HOMA-B: Homeostatic model assessment for B-cell function; HOMA-IR: Homeostatic model of assessment for insulin resistance; hs-CRP: Serum high sensitivity C-reactive protein; IL: Interleukin; JMCIS: Japanese version of the MCI Screen; MCI: Mild cognitive impairment; MDA: Malondialdehyde; MDS-UPDRS: Movement Disorders Society-Unified Parkinson’s Disease Rating Scale; MMDE: Mini mental state examination; MMSE: Mini-mental state examination; NA: Not available; NS: Non-significant; PD: Parkinson disease; PPAR-γ: Peroxisome proliferator-activated receptor gamma; PPAR-γ: Peroxisome proliferator-activated receptor gamma; POMS2: Profile of mood states 2nd edition; QOL: Quality of life; QUICKI: Quantitative insulin sensitivity check index; RBANS: Repeatable battery for the assessment of neuropsychological status; SBM: Spontaneous bowel movements; SD: Standard deviation; TAC: Total antioxidant capacity; TG: Serum triglycerides; TGF-β: Tumor necrosis factor beta; TNF-α: Tumor necrosis factor alpha; VLDL: Very low density lipoproteins.

## Data Availability

Not applicable.
